# The Interplay Between the Renin-Angiotensin-Aldosterone System and Parathyroid Hormone

**DOI:** 10.3389/fendo.2020.00539

**Published:** 2020-08-20

**Authors:** Ming-Hui Zheng, Fu-Xing-Zi Li, Feng Xu, Xiao Lin, Yi Wang, Qiu-Shuang Xu, Bei Guo, Ling-Qing Yuan

**Affiliations:** ^1^Hunan Provincial Key Laboratory of Metabolic Bone Diseases, Department of Endocrinology and Metabolism, National Clinical Research Center for Metabolic Disease, The Second Xiangya Hospital, Central South University, Changsha, China; ^2^Department of Radiology, The Second Xiangya Hospital, Central South University, Changsha, China

**Keywords:** renin-angiotensin-aldosterone system, aldosterone, angiotensin II, primary aldosteronism, parathyroid hormone, primary hyperparathyroidism

## Abstract

The renin-angiotensin-aldosterone system (RAAS) is the regulatory system by which renin induces aldosterone production. Angiotensin II (Ang II) is the main effector substance of the RAAS. The RAAS regulates blood pressure and electrolyte balance by controlling blood volume and peripheral resistance. Excessive activation of the RAAS is an important factor in the onset of cardiovascular disease and the deterioration of this disease. The most common RAAS abnormality is primary aldosteronism (PA). Parathyroid hormone (PTH) is a peptide secreted by the main cells of the parathyroid gland, which promotes elevated blood calcium (Ca^2+^) levels and decreased blood phosphorus (Pi) levels. Excessive secretion of PTH can cause primary hyperparathyroidism (PHPT). Parathyroidism is highly prevalent in postmenopausal women and is often associated with secondary osteoporosis. PA and PHPT are common endocrine system diseases. However, studies have shown a link between the RAAS and PTH, indicating a positive relationship between them. In this review, we explore the complex bidirectional relationship between the RAAS and PTH. We also point out possible future treatment options for related diseases based on this relationship.

## Introduction

Primary aldosteronism (PA) is often accompanied by primary hyperparathyroidism (PHPT) (1). In general, the higher the serum aldosterone concentration, the higher the serum Parathyroid hormone (PTH) concentration (2). Patients with PA often present with hypertension, and hypertension patients have a 2- to 8-fold higher risk of developing hyperparathyroidism as individuals with normal blood pressure (3). Hyperparathyroidism increases PTH levels and promotes bone absorption, which increases the risk of osteoporosis, indicating that the renin-angiotensin-aldosterone system (RAAS) is associated with osteoporosis. Meanwhile, RAAS can also cause osteoporosis by other mechanisms. Studies by Hatton et al. and Beavan et al. showed that bone may contain a tissue-renin-angiotensin-aldosterone system. Angiotensin I (Ang I), angiotensin II (Ang II), and aldosterone can effectively stimulate bone resorption of osteoclasts, causing osteolysis, thus leading to osteoporosis (4, 5). Mineralocorticoid receptor antagonism (MRA) may directly or indirectly, via PTH, affect human bone health (6).

It has been reported that PHPT patients have a higher prevalence of cardiovascular abnormalities than the general population (7, 8). PHPT leads to an increased prevalence of cardiovascular diseases and cardiovascular mortality (9–13). PTH is even used to predict cardiovascular risk (13, 14). The increase in cardiovascular mortality is associated with disturbances in the RAAS (15). PTH stimulates aldosterone secretion in human adrenocortical cells (12, 16, 17), which increases blood pressure. PTH can also raise blood pressure in five ways, thus increasing the risk of cardiovascular disease. Firstly, PTH may act as an ionophore for Calcium ion (Ca^2+^) and promote Ca^2+^ entry into cells, increasing vasoconstriction and raising blood pressure (18, 19). Secondly, PTH may promote vascular and cardiac remodeling, accelerating the development of cardiovascular disease in patients with PHPT (17, 20), but this is explained by the direct effect of PTH on vascular smooth muscle cells and endothelial cells, instead of the RAAS (21). Thirdly, higher PTH levels increase the degree of vascular stiffness and thereby increase cardiac afterload (22, 23). Fourthly, PTH can also increase plasma renin activity (PRA) and thereby increase blood pressure, while hypertension is associated with vascular calcification and aging (24). Moreover, the PRA level decreases to normal after parathyroidectomy (25, 26). Finally, elevated serum uric acid in PHPT patients may also lead to high blood pressure; the serum uric acid level falls significantly in patients with PHPT after parathyroidectomy (27, 28). Therefore, parathyroidectomy may reduce the risk of cardiovascular disease (11, 29, 30).

Considering that it is clinically important to find out whether a drug that affects one system will affect another system, thereby facilitating clinical diagnosis and treatment, it is interesting to discuss the complex relationship between the RAAS and PTH.

## Physiological Mechanism of RAAS Biomarkers and RAAS Inhibitors

The RAAS is an important body fluid regulation system in the human body and one of the most important regulators of sodium retention, potassium (K^+^) excretion, and blood volume and blood pressure. The RAAS has an important influence on cardiovascular hemodynamics and the development and progression of cardiovascular disease (31). RAAS includes renin, angiotensinogen, Ang I, angiotensin converting enzyme (ACE), Ang II, angiotensin II receptor 1 (AT1), aldosterone, and other components (32). Angiotensinogen is an alpha 2-globulin that is formed primarily by the liver and also by the kidney and other tissues. Renin is mainly formed and secreted by proximal glomerular epithelioid cells of afferent arterioles. The active form of renin has 340 amino acids (33). Decreased sodium intake, decreased extracellular fluid and blood volume, decreased arterial pressure, and increased sympathetic activity can stimulate renin release. Renin is an aspartic protease that cuts 10 peptides (Ang I) from angiotensinogen (34, 35). Renin is specific for angiotensinogen, and the activity of renin determines how much Ang I is produced (36, 37). Ang I is further cleaved to Ang II by ACE in pulmonary capillaries, endothelial cells, and renal epithelial cells (32, 35). Ang II can be converted to Ang III by removing aspartic acid from position 1 of the octapeptide. The role of angiotensin III is modest compared to the effect of angiotensin II (31). ACE2 is a monocarboxypeptidase that converts Ang I to Ang 1-9 and Ang II to Ang 1-7 (38).

Although the renin angiotensin system (RAS) contains multiple peptides, Ang II is the major active metabolite and acts on many different tissues and organs. Ang II promotes vasoconstriction and sympathetic enhancement and increases renal tubular reabsorption of sodium, leading to decreased blood flow and increased vascular resistance. Almost every organ system in the body responds to Ang II (37). Improper elevation of Ang II can lead to high blood pressure and increase the morbidity and mortality of cardiovascular disease (39). AT1 receptor (AT1R) is the primary receptor that mediates Ang II action in the heart and circulatory system (32). AT1R is widely distributed throughout the vasculature. The AT2 receptor (AT2R) can counteract the effects of the AT1R (31) and has a lower expression level in adults, but in some cardiovascular diseases, such as heart failure, the expression level of AT2R may increase.

Ang II and Ang III stimulate adrenal globular zone cells to secrete aldosterone, but Ang IV and Ang 1-7 do not induce adrenal aldosterone secretion. The subtype of angiotensin receptor they act on is not clear (40). Studies suggest that Ang II primarily stimulates aldosterone release by acting on AT1R (41). Mazzocchi et al. suggest that AT2R activation causes local release of catecholamines from chromaffin cells, which in turn enhances aldosterone secretion in a paracrine manner (42). Aldosterone promotes sodium retention and K^+^ excretion, retains water, and increases fluid volume (32). Excessive secretion of aldosterone causes PA (43). Excess aldosterone is associated with cardiovascular and kidney damage (inflammation, remodeling, and fibrosis) (44, 45). Aldosterone acts on the mineralocorticoid receptor (MR) of the kidney, thereby promoting tissue remodeling and angiogenesis (46).

**Figure 1:**
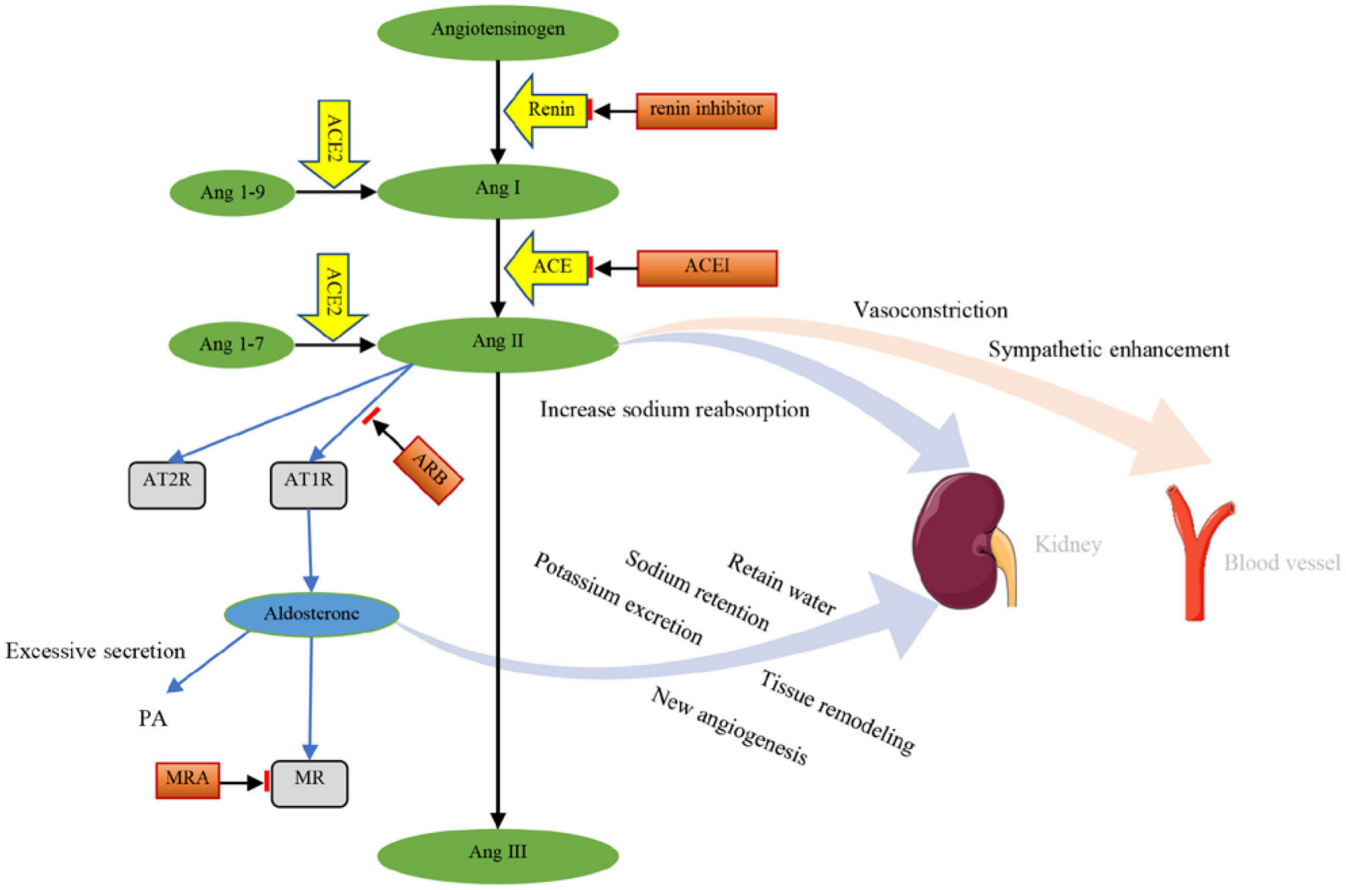
Components and effects of the RAAS. Renin cuts 10 peptides (Ang I) from angiotensinogen. The effect of renin can be inhibited by renin inhibitors. Ang I is further cleaved to Ang II by ACE. ACEI can inhibit the effect of ACE. Ang I and Ang II are converted into Ang1-9 and Ang1-7 by ACE2, respectively. Then, Ang II is converted into Ang III. Ang II acts on AT1R and AT2R. The effects of Ang II on AT1R can be blocked by ARB. Ang II promotes vasoconstriction and sympathetic enhancement and increases renal tubular reabsorption of sodium. Aldosterone retains water and promotes sodium retention, potassium excretion, tissue remodeling, and angiogenesis. Excessive secretion of aldosterone causes PA. Aldosterone acts on MR, which can be blocked by MRA. Ang I, angiotensin I; Ang II, angiotensin II; ACE, angiotensin converting enzyme; ACEI, angiotensin converting enzyme inhibitor; Ang1-9, angiotensin 1-9; Ang1-7, angiotensin 1-7; Ang III, angiotensin III; AT1R, angiotensin II receptor 1; AT2R, angiotensin II receptor 2; ARB, angiotensin receptor blocker; MR, mineralocorticoid receptor; MRA, mineralocorticoid receptor antagonism; PA, primary aldosteronism.

RAAS inhibitors include renin inhibitors, ACE inhibitors (ACEIs), AT1R antagonists, and MRAs (47). RAS inhibitors (RASIs) mainly contain the first three (44). ACEIs (ramipril, perindopril, captopril) control hypertension by inhibiting ACE to reduce the biosynthesis of Ang II (48). AT1R antagonists can block the action of Ang II on ATR1 and decrease aldosterone release (Figure 1) (40). RASIs inhibit the RAS, increase blood flow, and reduce peripheral vascular resistance, leading to decreased arterial pressure (37). Inhibition of RAS components has been successfully used to treat hypertension, heart failure, and end-organ damage (36). MRA (spironolactone, eplerenone) can be used to treat hypertension, PA, and peripheral edema associated with heart failure and other pathologies associated with aldosteronism (49). In addition, the combined use of ACEI, angiotensin receptor blocker (ARB), MRA, and other drugs can better improve heart failure, especially for patients with a reduced ejection fraction (50).

## Physiological Role of PTH

PTH is an 84 amino acid single-stranded peptide hormone secreted by the chief cells of the parathyroid gland. The active form of PTH (pth1-84) is inactivated via liver and kidney metabolism (plasma half-life is about 2–4 min) (51). The secretion of PTH is mainly regulated by the concentration of serum Ca^2+^ (52). When the serum Ca^2+^ concentration is low, the secretion of PTH increases. When the serum Ca^2+^ concentration is high, the secretion of PTH decreases (51). This relationship is mediated by the interaction between Ca^2+^ and the presence of calcium-sensitive receptors (CASR) on the surface of parathyroid cells (52–54). The serum phosphorus (Pi) level is another important factor regulating PTH secretion. Pi in serum can indirectly stimulate the proliferation of parathyroid cells and the secretion of PTH, thereby reducing the concentration of serum Ca^2+^ (55). Serum Pi can also directly enhance the secretion function of parathyroid cells by increasing the stability of PTH mRNA (56).

**Figure 2:**
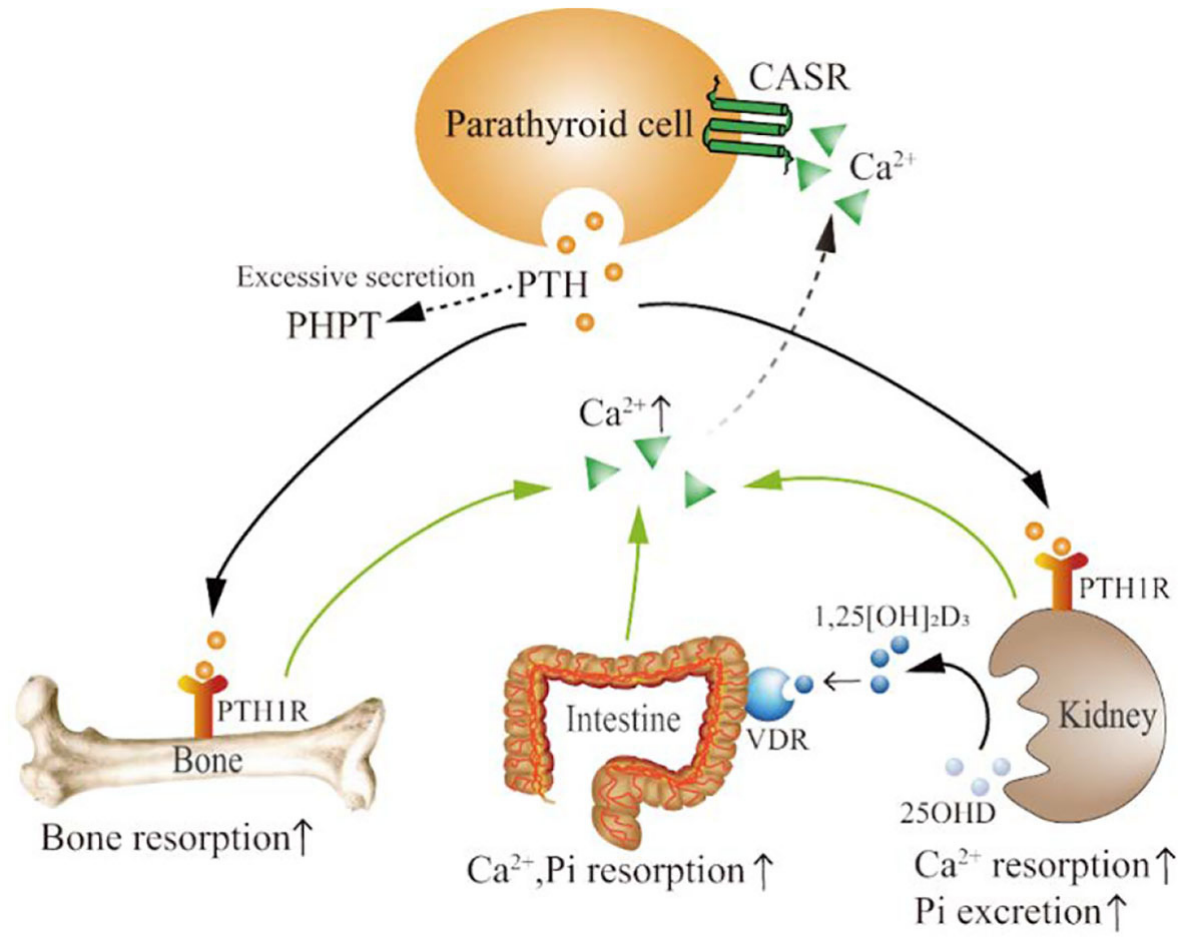
The physiological mechanism of PTH action. PTH is secreted by parathyroid cells. Excessive PTH secretion can cause PHPT. PTH functions by binding to the PTH1R in bone and kidney. PTH promotes bone absorption and the release of bone Ca^2+^, as well as the reabsorption of Ca^2+^ and the excretion of Pi in the kidney. PTH can also promote the conversion of 25OHD to 1,25[OH]_2_D_3_ in the kidney. 1,25[OH]_2_D_3_ binds to VDR, increasing the reabsorption of Ca^2+^ and Pi in the intestine. Serum Ca^2+^ regulates PTH secretion by binding to CASR on parathyroid cells. PTH, parathyroid hormone; PHPT, primary hyperparathyroidism; PTH1R, PTH1 receptor; Ca^2+^, calcium ions; Pi, phosphorus; 25OHD, 25-hydroxyvitamin D; 1,25[OH]_2_D_3_, 1,25-dihydroxyvitamin D; VDR, vitamin D receptor; CASR, calcium-sensitive receptors; ↑, upregulated.

The main target organs of PTH are the bones and kidneys (57, 58). PTH functions by binding to the receptor PTH/PTHrP receptor 1 (PTH1R) on target cells. PTH can mobilize bone Ca^2+^ into the blood and promote renal tubular reabsorption of Ca^2+^ and Pi excretion (54). PTH promotes the conversion of 25-hydroxyvitamin D (25OHD) in the kidney to 1,25-dihydroxyvitamin D (1,25[OH]_2_D_3_), which acts on the vitamin D receptor (VDR) to promote the intestinal reabsorption of Ca^2+^ and Pi, thereby increasing serum Ca^2+^ and Pi concentrations (Figure 2) (59, 60).

Parathyroid adenoma, hyperplasia, or cancer can secrete excessive amounts of PTH, causing PHPT (52). PHPT is the most common manifestation of abnormal PTH levels. In patients with PHPT, long-term increases in PTH levels can lead to bone lesions such as osteoporosis, fragility fractures, and bone pain, as well as kidney lesions such as kidney stones, kidney calcification, and reduced renal function (30, 43, 52, 57, 58). Long-term PHPT can cause a serious condition known as fibrocystic cysts, proximal muscle weakness due to type II fiber atrophy, and even neuropsychiatric symptoms (59). Parathyroidectomy is the most important treatment for PHPT. However, for patients who are not suitable for surgery or who refuse surgery, various drugs, such as bisphosphonates and calcimimetics, can be taken appropriately according to patient symptoms (61).

## Chronobiology and Chronotherapy of the RAAS/PTH

Blood pressure and biomarkers of RAAS have been shown to have circadian rhythms in humans (62); this circadian rhythm is also found in dogs (63). Studies have shown that the human circadian rhythm of RAAS is influenced by sodium intake (64), age, sex, and recumbency (65). Cugini et al. showed that limiting sodium intake amplifies the circadian rhythm of PRA and aldosterone. However, ACEIs inhibit this rhythm (66). The administration of ACEI at bedtime is more effective at controlling hypertension as compared to in the morning in hypertensive patients (67) and in a transverse aortic constriction (TAC) mouse model (68). Mochel et al. found that the timing of food intake is critical to the circadian rhythm of RAAS and blood pressure in dogs (69).

Similarly, the secretion of PTH is also rhythmic. In healthy humans, PTH is secreted primarily in a dual fashion. In addition to the most important tonic secretions, PTH can also be secreted in a low-amplitude and high-frequency pulse approximately every 20 min (70, 71). PTH release is affected by the blood Ca^2+^ concentration (72). This rhythmic secretion of PTH may be very important, as continuous administration of PTH leads to bone damage, while pulsed administration increases bone formation (73). Shinagawa et al. found that osteoporosis can be improved by simulating the pulsatile secretion of PTH to promote bone formation by oral administration of short-acting antagonists of CASR to rats (74).

Therefore, reference to the rhythmic secretion of RAAS and PTH has important diagnostic and therapeutic values for cardiovascular diseases or endocrine diseases in humans and animals.

## Effects of RAAS Biomarker Levels on PTH Levels

### How Does the RAAS Affect PTH

The RAAS mainly affects the secretion of PTH through AT1R and MR expressed in parathyroid tissue. AT1R and MR were detected in parathyroid adenoma tissue, with a 2- to 4-fold increase in expression when compared to normal parathyroid (75, 76). Chronic elevated aldosterone may increase PTH levels mainly by affecting renal function and serum Ca^2+^ (77). This can also be explained at the genetic level. Long-term injection of Ang II downregulates the expression of the Klotho gene in the kidney of rats (78), and downregulated Klotho expression will eventually cause an increase in PTH levels (79).

**Figure 3:**
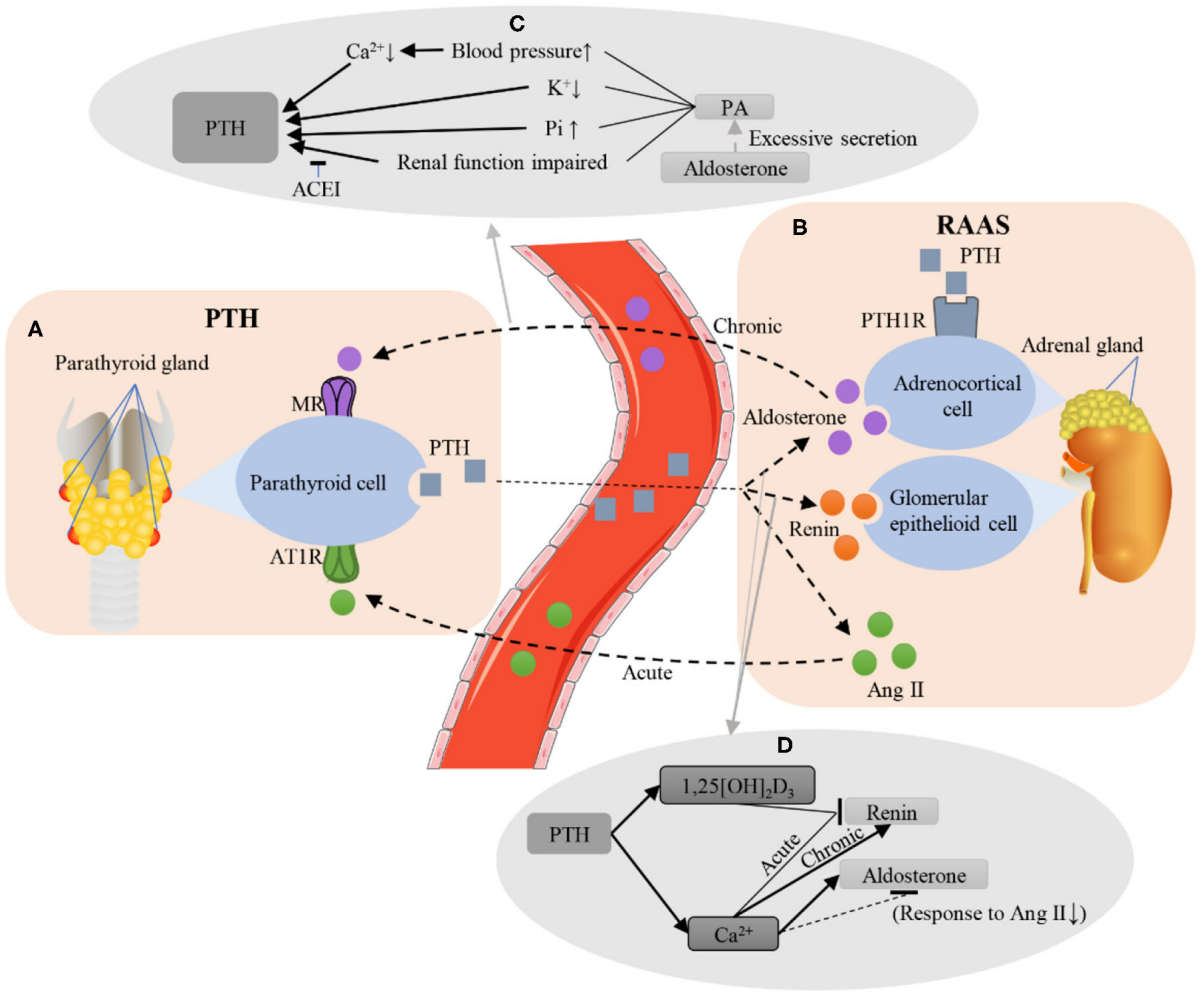
Interactions between the PTH and RAAS. **(A,B)** Brief illustration of RAAS and PTH. Under chronic conditions, the aldosterone secreted by the adrenal gland binds to the MR of the parathyroid gland. Under acute conditions, Ang II binds to AT1R in the parathyroid gland. PTH can bind to PTH1R in the adrenal gland to promote aldosterone secretion. In addition, PTH secretion can also increase the levels of renin and Ang II. **(C)** Several possible mechanisms for aldosterone to increase PTH levels. Excessive secretion of aldosterone causes PA, leading to the increase in blood pressure and serum Pi, the decrease in serum Ca^2+^ and K^+^, and the impairment of renal function, finally increasing the level of PTH, while ACEI can protect renal function and reduce PTH levels. **(D)** The possible mechanism of PTH regulating renin and aldosterone levels. PTH increased the serum levels of 1,25 [OH]_2_D_3_ and Ca^2+^. Acute elevation of Ca^2+^ and 1,25 [OH]_2_D_3_ inhibited renin secretion, while chronic elevation of Ca^2+^ could increase the renin level. Ca^2+^ can inhibit the diurnal decline of aldosterone, and it may also reduce the level of aldosterone by weakening the response of the kidney to Ang II. PTH, parathyroid hormone; RAAS, renin-angiotensin-aldosterone system; MR, mineralocorticoid receptor; Ang II, angiotensin II; AT1R, angiotensin II receptor 1; PTH1R, PTH1 receptor; PA, primary aldosteronism; Pi, phosphorus; Ca^2+^, calcium ions; K^+^, potassium; ACEI, angiotensin converting enzyme inhibitor; 1,25 [OH]_2_D_3_, 1,25-dihydroxyvitamin D. ↑, upregulated; ↓, downregulated.

The relationship between the RAAS and PTH is significantly positive, as patients with PA exhibit higher PTH levels compared to secondary aldosteronism and primary hypertension patients (2, 17, 77). Recent studies have suggested that mild hyperparathyroidism is a characteristic of PA (80), irrespective of the subtype of PA (81). Furthermore, PA may even contribute to secondary hyperparathyroidism (77). The regulatory effect of Ang II on PTH is influenced by the injection dose of Ang II and the adequacy status of vitamin D. Supplementation with vitamin D3 can enhance the secretion-promoting effect of low-dose Ang II (1 ng/kg/min for 90 min) on PTH. A high-dose infusion of Ang II (3 ng/kg/min for 90 min) leads to greater increases in aldosterone and a more robust PTH response. Under acute and chronic conditions, the components of the RAAS that affect PTH secretion may also be different. Brown et al. demonstrated that, under acute conditions, it is the acute increase in the Ang II level rather than the acute increase in the aldosterone level that increases the PTH level, indicating that Ang II may be an acute regulator of PTH (76). However, aldosterone may be a chronic regulator of PTH (Figures 3A,B). Studies have shown that chronic long-term increases in aldosterone levels can lead to an increase in PTH levels (2, 17, 77). Chhokar et al. and Rossi et al. reported that an aldosterone infusion led to hyperparathyroidism (82, 83). A study of 11 PA patients and 15 non-PA patients showed that the PTH level in PA patients was elevated and recovered after surgery or spironolactone treatment (43). Another study by Fischer et al. showed that a high aldosterone-to-renin ratio (ARR) was associated with high serum PTH concentrations in the general population, but they found no associations between serum aldosterone or renin and PTH levels (84).

### Changes in PTH After the Inhibition of RAAS Function

Several studies have shown that PTH levels return to normal after medical or surgical treatment in PA patients (81). A study by Mizobuchi et al. showed the lowering effect of enalapril on PTH levels in uremic mice (85). A series of human studies also reached similar conclusions. A multi-ethnic study of atherosclerosis showed that the use of ACEI reduces the level of aldosterone, thereby reducing the level of PTH. Moreover, RAAS inhibitor (RAASI) use decreased the level of mean PTH significantly than the use of non-RAASI antihypertensives. In addition, Ca^2+^-channel blockers were associated with higher PTH levels (2). In a single-arm pilot study, Zaheer et al. (86) found that 1 week of lisinopril therapy titrated to maximally tolerated blood pressure lowering resulted in a modest and marginally statistically significant lowering of PTH levels among participants with PHPT, but did not impact the level of PTH among participants without PHPT. However, there was a decrease in systolic blood pressure, an increase in PRA, and no changes in serum and urine Ca^2+^ in both groups. Studies showed that RASI use induced a lower PTH level in patients with continuous ambulatory peritoneal dialysis (CAPD) or end-stage renal failure (79, 87).

The use of ARB also significantly reduces the level of PTH (76, 87, 88), although this conclusion remains controversial. A study by Zaheer et al. demonstrated that chronic MRA use modestly lowered PTH levels and raised serum Ca^2+^ (86). Contrary to the above result, a randomized, placebo-controlled trial found that short-term low-dose valsartan (a kind of ARB) treatment affected neither PTH levels nor the aldosterone levels; however, valsartan increased renin levels (89). A randomized, placebo-controlled trial showed that the level of PTH was not decreased after treatment with eplerenone, a new aldosterone receptor antagonist. However, short-term (1–8 weeks) treatment with RAASI (ACEI and MRA) is unlikely to induce a robust and clinically meaningful reduction in PTH in patients with PHPT (90). Moreover, ACEIs and ARBs do not consistently inhibit the RAAS, which in turn may increase plasma aldosterone levels in some patients. This phenomenon is called “aldosterone escape” or “aldosterone breakthrough” (91). This phenomenon may explain why the use of ACEIs and ARB does not affect the level of PTH. Aldosteronoma resection decreases the PTH level (83). Adrenalectomy not only cures hyperaldosteronism but also corrects hyperparathyroidism (17, 80). Compared with MRA, surgical treatment reduces PTH levels more obviously in PA patients (77). However, adrenalectomy can cause short-term aldosterone deficiency, resulting in hyperkalemia (92), and can even cause adrenal insufficiency, such as Addison's disease, which is rare (93). The findings mentioned above provide convincing evidence for a causal relationship between the RAAS and PTH levels.

## The Level of RAAS Biomarker Changes With the Level of PTH

### How Does PTH Affect the RAAS

PTH receptor mRNA can be detected in the adrenal gland (94). PTH also autoradiographically binds to the adrenal cortex, suggesting that the adrenal gland is one of the target organs of PTH (95, 96). PHPT may lead to the inactivation of VDR and then increase the level of renin expression (97). The type 1 PTH receptor is expressed in the cytoplasm of aldosterone-producing adenoma cells and nodule cells, indicating that PTH could directly influence the synthesis of aldosterone (17, 75). PTH binds to PTH/PTH-related peptide receptors and voltage-gated l-type calcium channels to initiate multiple signal transduction pathways that activate the RAS (98). PTH can increase the release of cAMP and inositol triphosphate from adrenocortical cells through activating G-protein-coupled signaling cascades (99–101). PTH inhibitors have been shown to inhibit the production of cAMP by adrenocortical cells (95). Both adenylate cyclase inhibitors and phospholipase C blockers block the production of PTH. PTH may function through the PTH receptor coupled with PLC/PKC-dependent signaling cascades (102).

PTH has been demonstrated to have a positive effect on the RAAS (12, 99), and PTH can induce renin release (103–105). Saussine et al. reported that the renin stimulation of PTH may be mediated by the inhibition of calcium influx (106); however, a study by Hulter et al. showed that PTH infusion resulted in a significant transient increase in aldosterone excretion, but no significant changes in PRA were observed (107). Verheyen et al. proposed that the high incidence of arterial hypertension in PHPT may be partially explained by elevated aldosterone levels (108). PTH promotes aldosterone secretion in a dose-dependent manner with minimal and maximal effective concentrations of 10^−10^ M and 10^−8^ M (102); the stimulatory effect of PTH on aldosterone secretion is induced by Ca^2+^ (109). Jespersen et al. reported that serum Ang II levels are elevated in patients with PHPT (110). PTH alone or in combination with Ang II stimulates adrenal glomerular cells to secrete aldosterone as well as enhancing the role of Ang II in promoting aldosterone secretion (Figures 3A,B) (96). A study by Maniero et al. showed that PTH can trigger and/or maintain hyperaldosteronism in patients with secondary aldosteronism (17). Akmal et al. used a dog model of chronic renal failure (CRF) to cause secondary hyperparathyroidism to demonstrate that increased PTH levels can cause pulmonary hypertension (111), which has been shown to be related to RAAS disorders in humans (112).

Some studies have shown that PTH does not have a significant impact on the RAAS. Bernini et al. compared PRA in hyperparathyroidism patients vs. normal subjects and essential hypertensive (EH) patients, observing no activation of the RAAS, and only found a weak positive correlation between PTH and PRA (113). A study by Richards et al. showed that aldosterone levels in patients with PHPT were not elevated (Table 1) (117).

**Table 1:** Representative studies on the effects of PTH on aldosterone secretion.

**Study design**	**Subjects**	**Main conclusions**	**References**
Single-center, randomized, placebo-controlled, double-blind, parallel-armed cross-sectional study	136 patients diagnosed with PHPT	Hypertension in PHPT patients may be caused by elevated aldosterone levels	(108)
Case-control study	105 consecutive hypertensive patients, of whom 44 had PA and 61 had primary (essential) hypertension.	PTH can trigger and/or maintain hyperaldosteronism in patients with secondary aldosteronism	(17)
Clinical trial	8 patients with PA (6 hypertensive and 2 normotensive patients)	Aldosterone levels in patients with PHPT were not elevated	(113)
Clinical trial	34 patients with PHPT (10 hypertensive and 24 normotensive patients)	PTH stimulates aldosterone release, while parathyroidectomy reduces aldosterone levels	(26)
Clinical trial	16 patients with PHPT (13 females, 3 males)	PTH stimulates aldosterone release, while parathyroidectomy reduces aldosterone levels	(114)
Clinical trial	20 patients with PHPT, 26 EH patients, and 13 normotensives	There was no significant change in aldosterone levels in hyperparathyroidism patients, EH patients, and control group after parathyroid adenoma resection	(115)
Clinical trial	24 patients with PHPT, 16 patients with PHPT and EH, and 19 normal subjects	Parathyroidectomy did not change plasma aldosterone levels in hypertensive patients with PHPT and normal subjects	(116)
Experimental study	Dispersed adrenocortical cells obtained from adrenal glands removed from 16 consenting patients undergoing unilateral nephrectomy with ipsilateral adrenalectomy for renal cancer.	PTH promoted aldosterone secretion in a dose-dependent manner. PTH receptor antagonists can eliminate the secretory effect of PTH on aldosterone	(102)
Experimental study	Bovine adrenal glomerulosa cells	PTH alone or in combination with Ang II stimulates adrenal glomerular cells to secrete aldosterone	(96)

*PHPT, primary hyperparathyroidism; PTH, parathyroid hormone; Ang II, angiotensin II; PA, primary aldosterone; EH, essential hypertensive*.

Other studies have revealed a link between PTH1R and PRA. In the spontaneous hypertensive rat model driven by somatic human PTH1R (hPTH1R) gene expression, the overexpression of hPTH1R increases PRA, but the blood pressure remains unchanged (118). Interestingly, however, another study in adult rats in which PTH1R was overexpressed in the blood vessels, heart, kidneys, and other organs showed that PRA decreased 3 weeks after plasmid injection, thereby lowering blood pressure and heart rate in the rats (119). These rat models are very important for studying the function of PTH1R, because they have a high degree of genetic similarity with humans. Humans can not only accurately simulate human diseases in rat models but also guide the genetic modification of rats, so as to provide references for the treatment of human diseases.

### Changes in the RAAS After the Inhibition of Parathyroid Function

PTH receptor antagonists have been demonstrated to eliminate the secretory effect of PTH on aldosterone (102). Parathyroidectomy has been shown to reduce systolic and diastolic blood pressure in patients with PA and to improve metabolic complications associated with PHPT (120). However, the hypotensive effect of parathyroidectomy was only manifested in PHPT patients with hypertension (114), and this effect was not observed in patients with normal blood pressure (8). Conversely, Kovács et al. found that, in 16 patients with normal blood pressure in PHPT, removal of the parathyroid gland caused a significant decrease in systolic blood pressure (121). Diamond et al. reported that, after parathyroid surgery, systolic blood pressure decreased significantly in patients with normal blood pressure and hypertension (122). There are also studies showing that parathyroidectomy does not lower blood pressure (11, 123, 124). Studies by Kovács et al. and Gennari et al. showed that the direct effect of PTH on the RAAS may explain the role of parathyroidectomy on blood pressure (26, 121).

Studies by Kovács et al. (26), Gennari et al. (121), and Pacifici et al. (115) showed that PTH stimulates the release of aldosterone and that parathyroidectomy reduces blood pressure as well as plasma levels of renin and aldosterone. However, Bernini et al. demonstrated that PH patients are similar to EH patients regarding controls on PRA and aldosterone levels, and there was no significant change after parathyroid adenoma resection (113). Additionally, Salahudeen et al. found that parathyroidectomy did not alter PRA and plasma aldosterone levels in normotensive and hypertensive patients with PHPT (Table 1) (116). The above two studies indicate that PTH may not affect RAAS. Studies have also shown that PTH may have an indirect negative regulatory effect on the RAAS. Zawada et al. used a model of dogs with normal blood pressure and renovascular hypertension to confirm that thyroparathyroidectomy or chelation with EDTA to reduce blood Ca^2+^ will cause an increase in PRA (125).

## Discussion

The parathyroid gland expresses AT1R and MR, which supports the hypothesis that aldosterone contributes to the regulation of PTH secretion. The adrenal gland also expresses the type 1 PTH receptor, which likely explains why human adrenocortical cells respond with aldosterone and cortisol release to either PTH or PTH-related peptide (75). Studies have shown that the RAAS and PTH have a positive effect on each other.

There may be several other mechanisms that explain the increase in PTH as RAAS levels increase. Firstly, patients with PA often present with elevated blood pressure, and high blood pressure may alter the kidney's handling of Ca^2+^, which may lead to increased 24-h renal Ca^2+^ excretion and decreased serum Ca^2+^ (43, 126–129). Several studies have shown that the increase in the PTH level may be caused by a decrease in serum Ca^2+^ rather than a direct effect of aldosterone in patients with PA, as a low Ca^2+^ level may contribute to secondary hyperparathyroidism and eventually affect bone mass and strength (17, 77, 82, 88, 126, 130, 131). Secondly, a negative correlation between serum K^+^ and PTH levels has also been found (81). However, aldosterone can lower serum K^+^, so the stimulatory effect of aldosterone on PTH secretion may be mediated by K^+^. Thirdly, early impaired renal function in PA patients may cause parathyroid function enhancement (126), but the renoprotective effect may result in lower PTH levels (85). Enalapril reduces serum creatinine levels in rats, but enalapril reduces PTH levels through a protective effect on the kidney, rather than by directly acting on the parathyroid gland (85). Finally, changes in serum Pi levels in patients with PA and their effects on PTH levels also need to be considered (Figure 3C).

PTH exerts its effects on RAAS biomarkers through several possible mechanisms. PTH can increase the level of 1,25[OH] _2_D_3_ (59, 60), which will inhibit the expression of renin (97). Acute increases in plasma and extracellular and intracellular Ca^2+^ concentrations also inhibit renin secretion in renal proximal tubular cells, but long-term Ca^2+^ elevation can cause elevated PRA, which may be a compensation mechanism caused by Ca^2+^-mediated polyuria (132). PTH increases serum Ca^2+^ levels, and the increase in Ca^2+^ may inhibit the diurnal decline in PRA and aldosterone (133). In the case of hypercalcemia, the response of aldosterone to Ang II is weakened (134), which may explain why PTH inhibits the secretion of aldosterone levels (Figure 3D) (132).

As reviewed above, the interaction between RAAS and PTH-Calcium metabolism may be indirectly influenced by secondary factors such as electrolytes, blood pressure, and renal function. However, the direct interaction between the two systems is predominant. Further research is needed to investigate the complex interactions between PTH and RAAS and to assess whether PTH measurements can contribute to the diagnosis of PA. Elucidating the relationship between the RAAS and PTH has important clinical significance and future implications for the treatment of patients with PA or PHPT. RASIs might decrease PTH levels and improve these symptoms. MRA may reduce PTH-induced increases in the fracture rate in PA patients. However, the effect of MRA on PTH in patients without PA is unclear. MRAs may be a promising, novel, and inexpensive therapeutic option for PA patients with elevated PTH levels. Further studies are needed to investigate more medications that mitigate cardiovascular or skeletal diseases mediated by aldosterone and PTH.

## Author Contributions

L-QY: manuscript writing and approving final version of manuscript. M-HZ: study conduct, data analysis, and manuscript writing. FX, XL, YW, Q-SX, and BG: data analysis. All authors: reviewed the manuscript.

## Conflict of Interest

The authors declare that the research was conducted in the absence of any commercial or financial relationships that could be construed as a potential conflict of interest.
